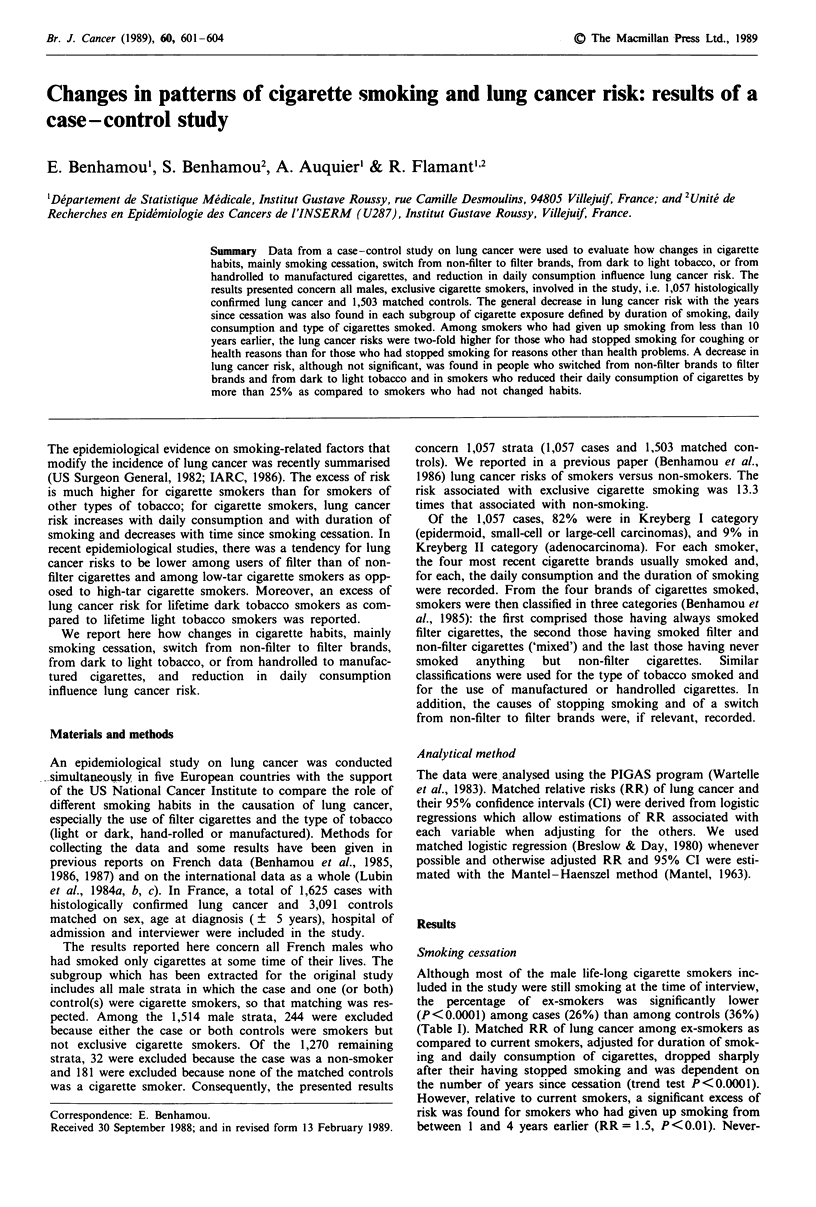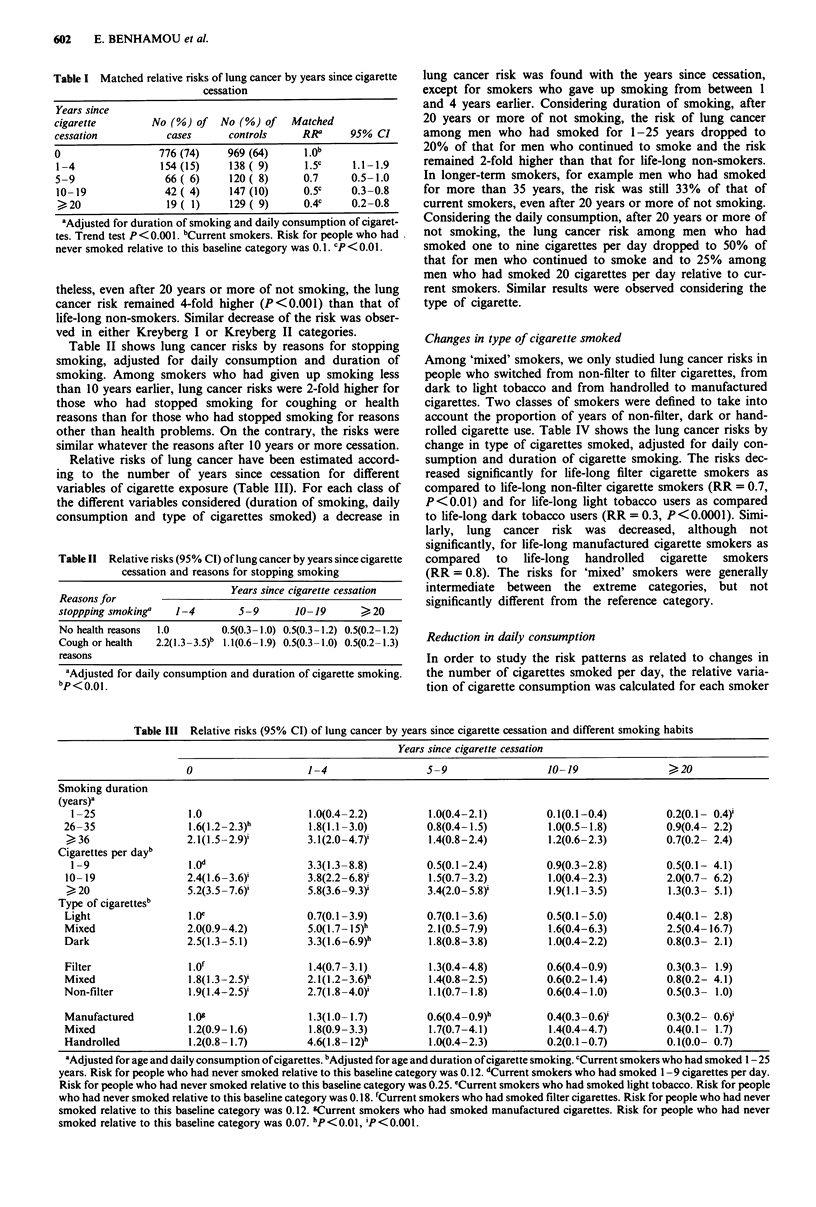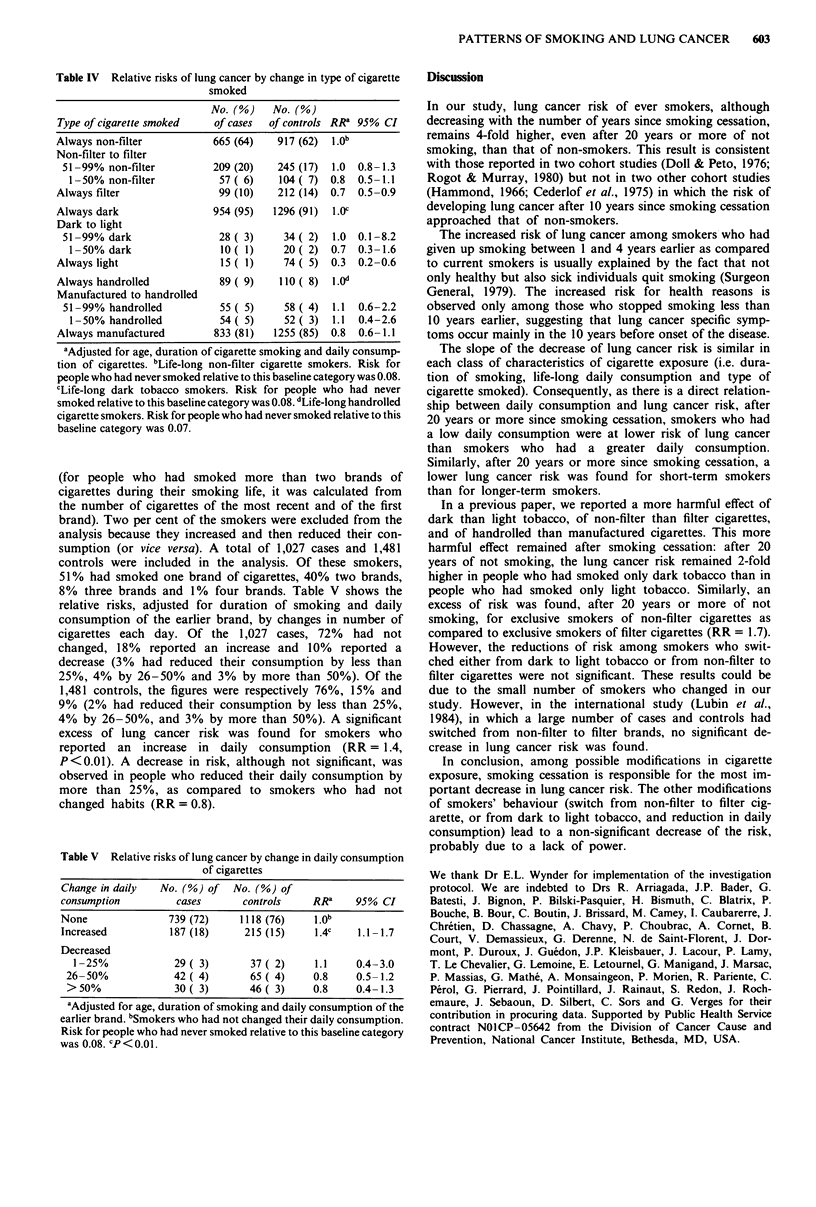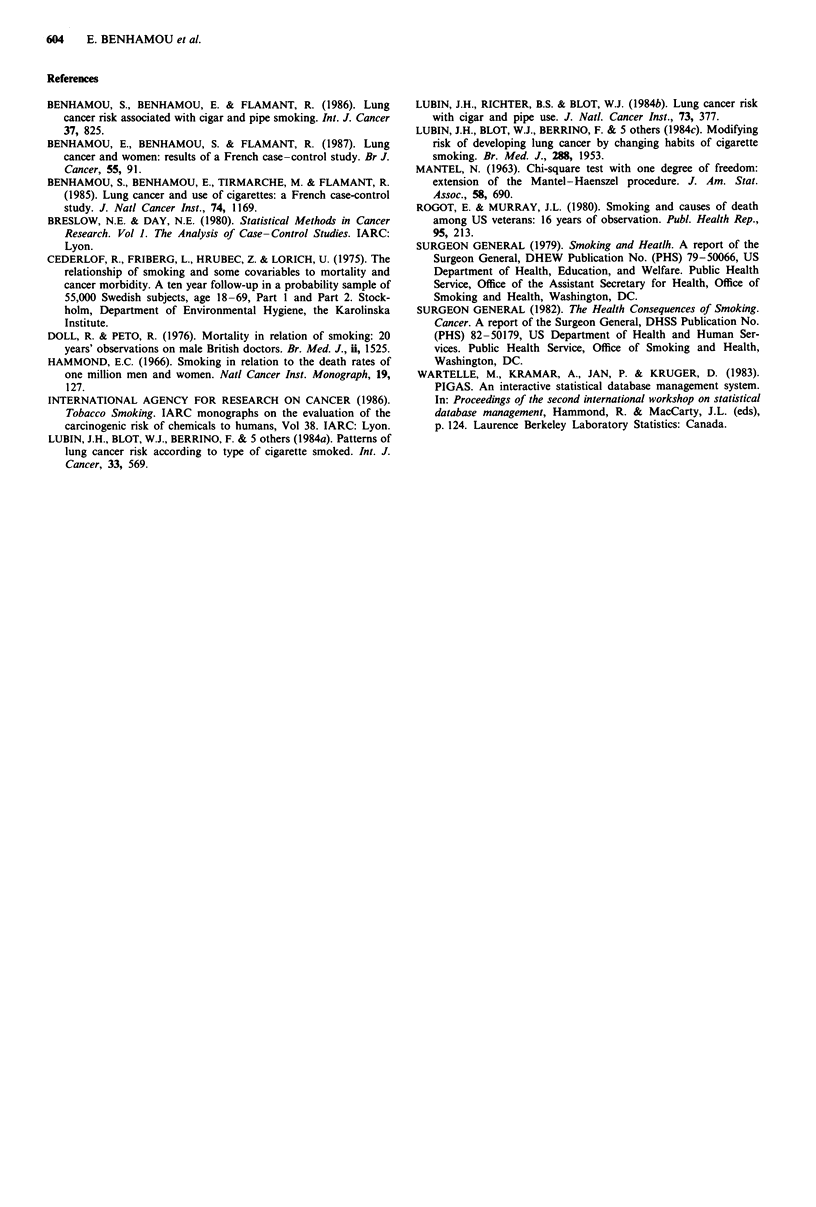# Changes in patterns of cigarette smoking and lung cancer risk: results of a case-control study.

**DOI:** 10.1038/bjc.1989.322

**Published:** 1989-10

**Authors:** E. Benhamou, S. Benhamou, A. Auquier, R. Flamant

**Affiliations:** DÃ©partement de Statistique MÃ©dicale, Institut Gustave Roussy, Villejuif, France.

## Abstract

Data from a case-control study on lung cancer were used to evaluate how changes in cigarette habits, mainly smoking cessation, switch from non-filter to filter brands, from dark to light tobacco, or from handrolled to manufactured cigarettes, and reduction in daily consumption influence lung cancer risk. The results presented concern all males, exclusive cigarette smokers, involved in the study, i.e. 1,057 histologically confirmed lung cancer and 1,503 matched controls. The general decrease in lung cancer risk with the years since cessation was also found in each subgroup of cigarette exposure defined by duration of smoking, daily consumption and type of cigarettes smoked. Among smokers who had given up smoking from less than 10 years earlier, the lung cancer risks were two-fold higher for those who had stopped smoking for coughing or health reasons than for those who had stopped smoking for reasons other than health problems. A decrease in lung cancer risk, although not significant, was found in people who switched from non-filter brands to filter brands and from dark to light tobacco and in smokers who reduced their daily consumption of cigarettes by more than 25% as compared to smokers who had not changed habits.


Br. J. Cancer (1989), 60, 601-604 © The Macmillan Press Ltd., 1989

### Changes in patterns of cigarette smoking and lung cancer risk: results of a case-control study

E. Benhamou[1], S. Benhamou[2], A. Auquier[1] & R. Flamant[1,2]

[1]*Département de Statistique Médicale, Institut Gustave Roussy, rue Camille Desmoulins, 94805 Villejuif, France; and* [2]*Unité de Recherches en Epidémiologie des Cancers de l'INSERM (U287), Institut Gustave Roussy, Villejuif, France.*

**Summary** Data from a case-control study on lung cancer were used to evaluate how changes in cigarette habits, mainly smoking cessation, switch from non-filter to filter brands, from dark to light tobacco, or from handrolled to manufactured cigarettes, and reduction in daily consumption influence lung cancer risk. The results presented concern all males, exclusive cigarette smokers, involved in the study, i.e. 1,057 histologically confirmed lung cancer and 1,503 matched controls. The general decrease in lung cancer risk with the years since cessation was also found in each subgroup of cigarette exposure defined by duration of smoking, daily consumption and type of cigarettes smoked. Among smokers who had given up smoking from less than 10 years earlier, the lung cancer risks were two-fold higher for those who had stopped smoking for coughing or health reasons than for those who had stopped smoking for reasons other than health problems. A decrease in lung cancer risk, although not significant, was found in people who switched from non-filter brands to filter brands and from dark to light tobacco and in smokers who reduced their daily consumption of cigarettes by more than 25% as compared to smokers who had not changed habits.

The epidemiological evidence on smoking-related factors that modify the incidence of lung cancer was recently summarised (US Surgeon General, 1982; IARC, 1986). The excess of risk is much higher for cigarette smokers than for smokers of other types of tobacco; for cigarette smokers, lung cancer risk increases with daily consumption and with duration of smoking and decreases with time since smoking cessation. In recent epidemiological studies, there was a tendency for lung cancer risks to be lower among users of filter than of non-filter cigarettes and among low-tar cigarette smokers as opposed to high-tar cigarette smokers. Moreover, an excess of lung cancer risk for lifetime dark tobacco smokers as compared to lifetime light tobacco smokers was reported.

We report here how changes in cigarette habits, mainly smoking cessation, switch from non-filter to filter brands, from dark to light tobacco, or from handrolled to manufactured cigarettes, and reduction in daily consumption influence lung cancer risk.

### Materials and methods

An epidemiological study on lung cancer was conducted simultaneously in five European countries with the support of the US National Cancer Institute to compare the role of different smoking habits in the causation of lung cancer, especially the use of filter cigarettes and the type of tobacco (light or dark, hand-rolled or manufactured). Methods for collecting the data and some results have been given in previous reports on French data (Benhamou *et al.*, 1985, 1986, 1987) and on the international data as a whole (Lubin *et al.*, 1984*a*, *b*, *c*). In France, a total of 1,625 cases with histologically confirmed lung cancer and 3,091 controls matched on sex, age at diagnosis (± 5 years), hospital of admission and interviewer were included in the study.

The results reported here concern all French males who had smoked only cigarettes at some time of their lives. The subgroup which has been extracted for the original study includes all male strata in which the case and one (or both) control(s) were cigarette smokers, so that matching was respected. Among the 1,514 male strata, 244 were excluded because either the case or both controls were smokers but not exclusive cigarette smokers. Of the 1,270 remaining strata, 32 were excluded because the case was a non-smoker and 181 were excluded because none of the matched controls was a cigarette smoker. Consequently, the presented results concern 1,057 strata (1,057 cases and 1,503 matched controls). We reported in a previous paper (Benhamou *et al.*, 1986) lung cancer risks of smokers versus non-smokers. The risk associated with exclusive cigarette smoking was 13.3 times that associated with non-smoking.

Of the 1,057 cases, 82% were in Kreyberg I category (epidermoid, small-cell or large-cell carcinomas), and 9% in Kreyberg II category (adenocarcinoma). For each smoker, the four most recent cigarette brands usually smoked and, for each, the daily consumption and the duration of smoking were recorded. From the four brands of cigarettes smoked, smokers were then classified in three categories (Benhamou *et al.*, 1985): the first comprised those having always smoked filter cigarettes, the second those having smoked filter and non-filter cigarettes ('mixed') and the last those having never smoked anything but non-filter cigarettes. Similar classifications were used for the type of tobacco smoked and for the use of manufactured or handrolled cigarettes. In addition, the causes of stopping smoking and of a switch from non-filter to filter brands were, if relevant, recorded.

### Analytical method

The data were analysed using the PIGAS program (Wartelle *et al.*, 1983). Matched relative risks (RR) of lung cancer and their 95% confidence intervals (CI) were derived from logistic regressions which allow estimations of RR associated with each variable when adjusting for the others. We used matched logistic regression (Breslow & Day, 1980) whenever possible and otherwise adjusted RR and 95% CI were estimated with the Mantel-Haenszel method (Mantel, 1963).

### Results

### Smoking cessation

Although most of the male life-long cigarette smokers included in the study were still smoking at the time of interview, the percentage of ex-smokers was significantly lower ($P<0.0001$) among cases (26%) than among controls (36%) (Table I). Matched RR of lung cancer among ex-smokers as compared to current smokers, adjusted for duration of smoking and daily consumption of cigarettes, dropped sharply after their having stopped smoking and was dependent on the number of years since cessation (trend test $P<0.0001$). However, relative to current smokers, a significant excess of risk was found for smokers who had given up smoking from between 1 and 4 years earlier (RR = 1.5, $P<0.01$). Never-

Correspondence: E. Benhamou.
Received 30 September 1988; and in revised form 13 February 1989.

**Table I** Matched relative risks of lung cancer by years since cigarette cessation

| *Years since cigarette cessation* | *No (%) of cases* | *No (%) of controls* | *Matched RR*[a] | *95% CI* |
|---|---|---|---|---|
| 0 | 776 (74) | 969 (64) | 1.0[b] | |
| 1-4 | 154 (15) | 138 ( 9) | 1.5[c] | 1.1-1.9 |
| 5-9 | 66 ( 6) | 120 ( 8) | 0.7 | 0.5-1.0 |
| 10-19 | 42 ( 4) | 147 (10) | 0.5[c] | 0.3-0.8 |
| ⩾20 | 19 ( 1) | 129 ( 9) | 0.4[c] | 0.2-0.8 |

[a]Adjusted for duration of smoking and daily consumption of cigarettes. Trend test $P<0.001$. [b]Current smokers. Risk for people who had never smoked relative to this baseline category was 0.1. [c]$P<0.01$.

theless, even after 20 years or more of not smoking, the lung cancer risk remained 4-fold higher ($P<0.001$) than that of life-long non-smokers. Similar decrease of the risk was observed in either Kreyberg I or Kreyberg II categories.

Table II shows lung cancer risks by reasons for stopping smoking, adjusted for daily consumption and duration of smoking. Among smokers who had given up smoking less than 10 years earlier, lung cancer risks were 2-fold higher for those who had stopped smoking for coughing or health reasons than for those who had stopped smoking for reasons other than health problems. On the contrary, the risks were similar whatever the reasons after 10 years or more cessation.

Relative risks of lung cancer have been estimated according to the number of years since cessation for different variables of cigarette exposure (Table III). For each class of the different variables considered (duration of smoking, daily consumption and type of cigarettes smoked) a decrease in lung cancer risk was found with the years since cessation, except for smokers who gave up smoking from between 1 and 4 years earlier. Considering duration of smoking, after 20 years or more of not smoking, the risk of lung cancer among men who had smoked for 1-25 years dropped to 20% of that for men who continued to smoke and the risk remained 2-fold higher than that for life-long non-smokers. In longer-term smokers, for example men who had smoked for more than 35 years, the risk was still 33% of that of current smokers, even after 20 years or more of not smoking. Considering the daily consumption, after 20 years or more of not smoking, the lung cancer risk among men who had smoked one to nine cigarettes per day dropped to 50% of that for men who continued to smoke and to 25% among men who had smoked 20 cigarettes per day relative to current smokers. Similar results were observed considering the type of cigarette.

**Table II** Relative risks (95% CI) of lung cancer by years since cigarette cessation and reasons for stopping smoking

| *Reasons for stoppping smoking*[a] | *Years since cigarette cessation* 1-4 | 5-9 | 10-19 | ⩾20 |
|---|---|---|---|---|
| No health reasons | 1.0 | 0.5(0.3-1.0) | 0.5(0.3-1.2) | 0.5(0.2-1.2) |
| Cough or health reasons | 2.2(1.3-3.5)[b] | 1.1(0.6-1.9) | 0.5(0.3-1.0) | 0.5(0.2-1.3) |

[a]Adjusted for daily consumption and duration of cigarette smoking. [b]$P<0.01$.

### *Changes in type of cigarette smoked*

Among 'mixed' smokers, we only studied lung cancer risks in people who switched from non-filter to filter cigarettes, from dark to light tobacco and from handrolled to manufactured cigarettes. Two classes of smokers were defined to take into account the proportion of years of non-filter, dark or handrolled cigarette use. Table IV shows the lung cancer risks by change in type of cigarettes smoked, adjusted for daily consumption and duration of cigarette smoking. The risks decreased significantly for life-long filter cigarette smokers as compared to life-long non-filter cigarette smokers (RR = 0.7, $P<0.01$) and for life-long light tobacco users as compared to life-long dark tobacco users (RR = 0.3, $P<0.0001$). Similarly, lung cancer risk was decreased, although not significantly, for life-long manufactured cigarette smokers as compared to life-long handrolled cigarette smokers (RR = 0.8). The risks for 'mixed' smokers were generally intermediate between the extreme categories, but not significantly different from the reference category.

### *Reduction in daily consumption*

In order to study the risk patterns as related to changes in the number of cigarettes smoked per day, the relative variation of cigarette consumption was calculated for each smoker

**Table III** Relative risks (95% CI) of lung cancer by years since cigarette cessation and different smoking habits

| | *Years since cigarette cessation* 0 | 1-4 | 5-9 | 10-19 | ⩾20 |
|---|---|---|---|---|---|
| Smoking duration (years)[a] | | | | | |
| 1-25 | 1.0 | 1.0(0.4-2.2) | 1.0(0.4-2.1) | 0.1(0.1-0.4) | 0.2(0.1- 0.4)[i] |
| 26-35 | 1.6(1.2-2.3)[h] | 1.8(1.1-3.0) | 0.8(0.4-1.5) | 1.0(0.5-1.8) | 0.9(0.4- 2.2) |
| ⩾36 | 2.1(1.5-2.9)[i] | 3.1(2.0-4.7)[i] | 1.4(0.8-2.4) | 1.2(0.6-2.3) | 0.7(0.2- 2.4) |
| Cigarettes per day[b] | | | | | |
| 1-9 | 1.0[d] | 3.3(1.3-8.8) | 0.5(0.1-2.4) | 0.9(0.3-2.8) | 0.5(0.1- 4.1) |
| 10-19 | 2.4(1.6-3.6)[i] | 3.8(2.2-6.8)[i] | 1.5(0.7-3.2) | 1.0(0.4-2.3) | 2.0(0.7- 6.2) |
| ⩾20 | 5.2(3.5-7.6)[i] | 5.8(3.6-9.3)[i] | 3.4(2.0-5.8)[i] | 1.9(1.1-3.5) | 1.3(0.3- 5.1) |
| Type of cigarettes[b] | | | | | |
| Light | 1.0[e] | 0.7(0.1-3.9) | 0.7(0.1-3.6) | 0.5(0.1-5.0) | 0.4(0.1- 2.8) |
| Mixed | 2.0(0.9-4.2) | 5.0(1.7-15)[h] | 2.1(0.5-7.9) | 1.6(0.4-6.3) | 2.5(0.4-16.7) |
| Dark | 2.5(1.3-5.1) | 3.3(1.6-6.9)[h] | 1.8(0.8-3.8) | 1.0(0.4-2.2) | 0.8(0.3- 2.1) |
| Filter | 1.0[f] | 1.4(0.7-3.1) | 1.3(0.4-4.8) | 0.6(0.4-0.9) | 0.3(0.3- 1.9) |
| Mixed | 1.8(1.3-2.5)[i] | 2.1(1.2-3.6)[h] | 1.4(0.8-2.5) | 0.6(0.2-1.4) | 0.8(0.2- 4.1) |
| Non-filter | 1.9(1.4-2.5)[i] | 2.7(1.8-4.0)[i] | 1.1(0.7-1.8) | 0.6(0.4-1.0) | 0.5(0.3- 1.0) |
| Manufactured | 1.0[g] | 1.3(1.0-1.7) | 0.6(0.4-0.9)[h] | 0.4(0.3-0.6)[i] | 0.3(0.2- 0.6)[i] |
| Mixed | 1.2(0.9-1.6) | 1.8(0.9-3.3) | 1.7(0.7-4.1) | 1.4(0.4-4.7) | 0.4(0.1- 1.7) |
| Handrolled | 1.2(0.8-1.7) | 4.6(1.8-12)[h] | 1.0(0.4-2.3) | 0.2(0.1-0.7) | 0.1(0.0- 0.7) |

[a]Adjusted for age and daily consumption of cigarettes. [b]Adjusted for age and duration of cigarette smoking. [c]Current smokers who had smoked 1-25 years. Risk for people who had never smoked relative to this baseline category was 0.12. [d]Current smokers who had smoked 1-9 cigarettes per day. Risk for people who had never smoked relative to this baseline category was 0.25. [e]Current smokers who had smoked light tobacco. Risk for people who had never smoked relative to this baseline category was 0.18. [f]Current smokers who had smoked filter cigarettes. Risk for people who had never smoked relative to this baseline category was 0.12. [g]Current smokers who had smoked manufactured cigarettes. Risk for people who had never smoked relative to this baseline category was 0.07. [h]$P<0.01$, [i]$P<0.001$.

**Table IV** Relative risks of lung cancer by change in type of cigarette smoked

| *Type of cigarette smoked* | *No. (%) of cases* | *No. (%) of controls* | *RR*[a] | *95% CI* |
|---|---|---|---|---|
| Always non-filter | 665 (64) | 917 (62) | 1.0[b] | |
| Non-filter to filter | | | | |
| 51–99% non-filter | 209 (20) | 245 (17) | 1.0 | 0.8–1.3 |
| 1–50% non-filter | 57 ( 6) | 104 ( 7) | 0.8 | 0.5–1.1 |
| Always filter | 99 (10) | 212 (14) | 0.7 | 0.5–0.9 |
| Always dark | 954 (95) | 1296 (91) | 1.0[c] | |
| Dark to light | | | | |
| 51–99% dark | 28 ( 3) | 34 ( 2) | 1.0 | 0.1–8.2 |
| 1–50% dark | 10 ( 1) | 20 ( 2) | 0.7 | 0.3–1.6 |
| Always light | 15 ( 1) | 74 ( 5) | 0.3 | 0.2–0.6 |
| Always handrolled | 89 ( 9) | 110 ( 8) | 1.0[d] | |
| Manufactured to handrolled | | | | |
| 51–99% handrolled | 55 ( 5) | 58 ( 4) | 1.1 | 0.6–2.2 |
| 1–50% handrolled | 54 ( 5) | 52 ( 3) | 1.1 | 0.4–2.6 |
| Always manufactured | 833 (81) | 1255 (85) | 0.8 | 0.6–1.1 |

[a]Adjusted for age, duration of cigarette smoking and daily consumption of cigarettes. [b]Life-long non-filter cigarette smokers. Risk for people who had never smoked relative to this baseline category was 0.08. [c]Life-long dark tobacco smokers. Risk for people who had never smoked relative to this baseline category was 0.08. [d]Life-long handrolled cigarette smokers. Risk for people who had never smoked relative to this baseline category was 0.07.

(for people who had smoked more than two brands of cigarettes during their smoking life, it was calculated from the number of cigarettes of the most recent and of the first brand). Two per cent of the smokers were excluded from the analysis because they increased and then reduced their consumption (or *vice versa*). A total of 1,027 cases and 1,481 controls were included in the analysis. Of these smokers, 51% had smoked one brand of cigarettes, 40% two brands, 8% three brands and 1% four brands. Table V shows the relative risks, adjusted for duration of smoking and daily consumption of the earlier brand, by changes in number of cigarettes each day. Of the 1,027 cases, 72% had not changed, 18% reported an increase and 10% reported a decrease (3% had reduced their consumption by less than 25%, 4% by 26–50% and 3% by more than 50%). Of the 1,481 controls, the figures were respectively 76%, 15% and 9% (2% had reduced their consumption by less than 25%, 4% by 26–50%, and 3% by more than 50%). A significant excess of lung cancer risk was found for smokers who reported an increase in daily consumption (RR = 1.4, $P < 0.01$). A decrease in risk, although not significant, was observed in people who reduced their daily consumption by more than 25%, as compared to smokers who had not changed habits (RR = 0.8).

**Table V** Relative risks of lung cancer by change in daily consumption of cigarettes

| *Change in daily consumption* | *No. (%) of cases* | *No. (%) of controls* | *RR*[a] | *95% CI* |
|---|---|---|---|---|
| None | 739 (72) | 1118 (76) | 1.0[b] | |
| Increased | 187 (18) | 215 (15) | 1.4[c] | 1.1–1.7 |
| Decreased | | | | |
| 1–25% | 29 ( 3) | 37 ( 2) | 1.1 | 0.4–3.0 |
| 26–50% | 42 ( 4) | 65 ( 4) | 0.8 | 0.5–1.2 |
| >50% | 30 ( 3) | 46 ( 3) | 0.8 | 0.4–1.3 |

[a]Adjusted for age, duration of smoking and daily consumption of the earlier brand. [b]Smokers who had not changed their daily consumption. Risk for people who had never smoked relative to this baseline category was 0.08. [c]$P < 0.01$.

### Discussion

In our study, lung cancer risk of ever smokers, although decreasing with the number of years since smoking cessation, remains 4-fold higher, even after 20 years or more of not smoking, than that of non-smokers. This result is consistent with those reported in two cohort studies (Doll & Peto, 1976; Rogot & Murray, 1980) but not in two other cohort studies (Hammond, 1966; Cederlof *et al.*, 1975) in which the risk of developing lung cancer after 10 years since smoking cessation approached that of non-smokers.

The increased risk of lung cancer among smokers who had given up smoking between 1 and 4 years earlier as compared to current smokers is usually explained by the fact that not only healthy but also sick individuals quit smoking (Surgeon General, 1979). The increased risk for health reasons is observed only among those who stopped smoking less than 10 years earlier, suggesting that lung cancer specific symptoms occur mainly in the 10 years before onset of the disease.

The slope of the decrease of lung cancer risk is similar in each class of characteristics of cigarette exposure (i.e. duration of smoking, life-long daily consumption and type of cigarette smoked). Consequently, as there is a direct relationship between daily consumption and lung cancer risk, after 20 years or more since smoking cessation, smokers who had a low daily consumption were at lower risk of lung cancer than smokers who had a greater daily consumption. Similarly, after 20 years or more since smoking cessation, a lower lung cancer risk was found for short-term smokers than for longer-term smokers.

In a previous paper, we reported a more harmful effect of dark than light tobacco, of non-filter than filter cigarettes, and of handrolled than manufactured cigarettes. This more harmful effect remained after smoking cessation: after 20 years of not smoking, the lung cancer risk remained 2-fold higher in people who had smoked only dark tobacco than in people who had smoked only light tobacco. Similarly, an excess of risk was found, after 20 years or more of not smoking, for exclusive smokers of non-filter cigarettes as compared to exclusive smokers of filter cigarettes (RR = 1.7). However, the reductions of risk among smokers who switched either from dark to light tobacco or from non-filter to filter cigarettes were not significant. These results could be due to the small number of smokers who changed in our study. However, in the international study (Lubin *et al.*, 1984), in which a large number of cases and controls had switched from non-filter to filter brands, no significant decrease in lung cancer risk was found.

In conclusion, among possible modifications in cigarette exposure, smoking cessation is responsible for the most important decrease in lung cancer risk. The other modifications of smokers' behaviour (switch from non-filter to filter cigarette, or from dark to light tobacco, and reduction in daily consumption) lead to a non-significant decrease of the risk, probably due to a lack of power.

We thank Dr E.L. Wynder for implementation of the investigation protocol. We are indebted to Drs R. Arriagada, J.P. Bader, G. Batesti, J. Bignon, P. Bilski-Pasquier, H. Bismuth, C. Blatrix, P. Bouche, B. Bour, C. Boutin, J. Brissard, M. Camey, I. Caubarerre, J. Chrétien, D. Chassagne, A. Chavy, P. Choubrac, A. Cornet, B. Court, V. Demassieux, G. Derenne, N. de Saint-Florent, J. Dormont, P. Duroux, J. Guédon, J.P. Kleisbauer, J. Lacour, P. Lamy, T. Le Chevalier, G. Lemoine, E. Letournel, G. Manigand, J. Marsac, P. Massias, G. Mathé, A. Monsaingeon, P. Morien, R. Pariente, C. Pérol, G. Pierrard, J. Pointillard, J. Rainaut, S. Redon, J. Rochemaure, J. Sebaoun, D. Silbert, C. Sors and G. Verges for their contribution in procuring data. Supported by Public Health Service contract N01CP-05642 from the Division of Cancer Cause and Prevention, National Cancer Institute, Bethesda, MD, USA.

### References

BENHAMOU, S., BENHAMOU, E. & FLAMANT, R. (1986). Lung cancer risk associated with cigar and pipe smoking. *Int. J. Cancer* **37**, 825.

BENHAMOU, E., BENHAMOU, S. & FLAMANT, R. (1987). Lung cancer and women: results of a French case-control study. *Br J. Cancer*, **55**, 91.

BENHAMOU, S., BENHAMOU, E., TIRMARCHE, M. & FLAMANT, R. (1985). Lung cancer and use of cigarettes: a French case-control study. *J. Natl Cancer Inst.*, **74**, 1169.

BRESLOW, N.E. & DAY, N.E. (1980). *Statistical Methods in Cancer Research. Vol 1. The Analysis of Case-Control Studies*. IARC: Lyon.

CEDERLOF, R., FRIBERG, L., HRUBEC, Z. & LORICH, U. (1975). The relationship of smoking and some covariables to mortality and cancer morbidity. A ten year follow-up in a probability sample of 55,000 Swedish subjects, age 18-69, Part 1 and Part 2. Stockholm, Department of Environmental Hygiene, the Karolinska Institute.

DOLL, R. & PETO, R. (1976). Mortality in relation of smoking: 20 years' observations on male British doctors. *Br. Med. J.*, **ii**, 1525.

HAMMOND, E.C. (1966). Smoking in relation to the death rates of one million men and women. *Natl Cancer Inst. Monograph*, **19**, 127.

INTERNATIONAL AGENCY FOR RESEARCH ON CANCER (1986). *Tobacco Smoking*. IARC monographs on the evaluation of the carcinogenic risk of chemicals to humans, Vol 38. IARC: Lyon.

LUBIN, J.H., BLOT, W.J., BERRINO, F. & 5 others (1984*a*). Patterns of lung cancer risk according to type of cigarette smoked. *Int. J. Cancer*, **33**, 569.

LUBIN, J.H., RICHTER, B.S. & BLOT, W.J. (1984*b*). Lung cancer risk with cigar and pipe use. *J. Natl. Cancer Inst.*, **73**, 377.

LUBIN, J.H., BLOT, W.J., BERRINO, F. & 5 others (1984*c*). Modifying risk of developing lung cancer by changing habits of cigarette smoking. *Br. Med. J.*, **288**, 1953.

MANTEL, N. (1963). Chi-square test with one degree of freedom: extension of the Mantel-Haenszel procedure. *J. Am. Stat. Assoc.*, **58**, 690.

ROGOT, E. & MURRAY, J.L. (1980). Smoking and causes of death among US veterans: 16 years of observation. *Publ. Health Rep.*, **95**, 213.

SURGEON GENERAL (1979). *Smoking and Heatlh*. A report of the Surgeon General, DHEW Publication No. (PHS) 79-50066, US Department of Health, Education, and Welfare. Public Health Service, Office of the Assistant Secretary for Health, Office of Smoking and Health, Washington, DC.

SURGEON GENERAL (1982). *The Health Consequences of Smoking. Cancer*. A report of the Surgeon General, DHSS Publication No. (PHS) 82-50179, US Department of Health and Human Services. Public Health Service, Office of Smoking and Health, Washington, DC.

WARTELLE, M., KRAMAR, A., JAN, P. & KRUGER, D. (1983). PIGAS. An interactive statistical database management system. In: *Proceedings of the second international workshop on statistical database management*, Hammond, R. & MacCarty, J.L. (eds), p. 124. Laurence Berkeley Laboratory Statistics: Canada.